# Cervical Cancer Screening in Women With Physical Disabilities

**DOI:** 10.1001/jamanetworkopen.2024.57290

**Published:** 2025-01-29

**Authors:** Alexandra H. Vinson, Corrianne Norrid, Elizabeth K. Haro, Susan Ernst, Christelle El Khoury, Martha L. Alves, Autumn Kieber-Emmons, Ashwini Kamath Mulki, Emma A. Butcher, Claire Kalpakjian, Michael M. McKee, Diane M. Harper

**Affiliations:** 1 Department of Learning Health Sciences, University of Michigan, Ann Arbor; 2 Short Term Biomedical Research Training Program, University of Michigan, Ann Arbor; 3 Department of Family Medicine, University of Michigan, Ann Arbor; 4 University Health Systems, University of Michigan, Ann Arbor; 5 Department of Family Medicine, Lehigh Valley Health Network, Allentown, Pennsylvania; 6 Department of Physical Medicine and Rehabilitation, University of Michigan, Ann Arbor; 7 Department of Obstetrics and Gynecology, University of Michigan, Ann Arbor; 8 Department of Obstetrics and Gynecology, University of Michigan, Ann Arbor; 9 Department of Women’s and Gender Studies, University of Michigan, Ann Arbor; 10 Department of Bioengineering, University of Michigan, Ann Arbor

## Abstract

**Importance:**

Cervical cancer screening is a crucial public health intervention, but screening disparities exist for women with physical disabilities (WWPD).

**Objective:**

To explore the experiences of WWPD with both traditional speculum examination–based screening and at-home self-sampling for cervical cancer screening.

**Design, Setting, and Participants:**

This qualitative study enrolled 56 WWPD to test self-sampling kits, provide feedback via a survey, and participate in a qualitative interview. An interprofessional team conducted semistructured interviews with 16 key informants and 40 pilot participants for 56 WWPD from November 1, 2021, through April 30, 2023. All completed a self-administered quantitative survey. Key informants’ experiences with 4 self-sampling devices helped determine which 2 self-sampling kits would be offered to participants in a pilot study.

**Main Outcomes and Measures:**

A coding scheme was developed to represent inductive codes generated through preliminary coding and deductive codes representing domains from the Theoretical Domains Framework. This coding scheme was used to conduct a 2-pass thematic analysis.

**Results:**

Of the 56 WWPD (mean [SD] age, 45.4 [9.1] years) who participated in the study, 28 (50.0%) were up to date with cervical cancer screening. Participants described accessibility barriers and clinician ableism that made speculum-based in-office examinations difficult, leading some participants to delay or avoid screening. In contrast, participants described self-screening as more comfortable and convenient, regardless of whether they prefer future speculum-based screening. Their responses also allowed for the exploration of how screening preferences may impact future screening behavior.

**Conclusions and Relevance:**

Interviews with WWPD suggested that access to self-sampling screening options would be more comfortable for cervical cancer screening participation. Understanding participants’ experiences with self-sampling devices generates insights into improving screening experiences for WWPD.

## Key Points

**Question:**

How can the cervical cancer screening experiences of women with physical disabilities (WWPD) be improved?

**Findings:**

In this qualitative study of 56 WWPD, those who face disability-specific barriers from the in-office physician-led speculum examination shared their experiences testing self-screening devices for cervical cancer screening through qualitative interviews. WWPD reported that self-sampling options would be comfortable and convenient for future screening.

**Meaning:**

Interviews with WWPD indicated that access to self-sampling options would be more comfortable for cervical cancer screening participation, findings that can inform the promotion of recently US Food and Drug Administration–approved self-sampling devices.

## Teaser

This qualitative study explores the experiences of women with physical disabilities with at-home self-sampling options for cervical cancer screening compared with traditional speculum examination–based screening.

## Introduction

Cervical cancer screening detects nearly half of all cervical cancer cases annually.^1^ Unfortunately, women with physical disabilities (WWPD) are 48% less likely to receive screening than women without physical disabilities.^2^ Existing research suggests that contributors to inadequate screening among WWPD include lack of accessibility,^3^ lack of cultural competency among health care professionals,^4^ and assumptions that WWPD are not sexually active and therefore do not require screening.^5,6^ The proportion of women with disabilities increases yearly, with physical disabilities being the most common at 51% of the population with disabilities.^7^

Human papillomavirus (HPV), the cause of nearly all cervical cancers, is primarily transmitted sexually through skin-to-skin contact.^8,9^ Because it is noninferior in sensitivity and specificity to the speculum-based examination, self-screening for primary HPV has been implemented in many European countries as an alternative screening method for cervical cancer.^10^ Using self-sampling kits increased participation among those least likely to screen.^10^ Self-screening can occur at home, where individuals can control timing, positioning, and privacy.

In the US, WWPD have not been engaged in public health work to expand access and design cervical screening services that meet their needs. Moreover, establishing the prevalence of cervical cancer screening for WWPD is limited to analyzing self-report survey items from the National Health Interview Survey (NHIS), the Medical Expenditure Panel Survey (MEPS), the Health Information National Trends Survey, or the Behavioral Risk Factor and Surveillance System (BRFSS).^11^ Despite knowing that WWPD are less likely to be screened,^12^ survey formats do not provide WWPD an opportunity to speak freely about concerns not anticipated by survey response choices, compounding a situation where their voices do not inform public health measures. This study explores WWPD’s screening experiences with novel vaginal and urine self-sampling devices designed for primary HPV screening for cervical cancer, WWPDs’ prior speculum-based cervical cancer screening experiences, and how a self-sampling option may reduce barriers and increase intent to screen.

## Methods

This qualitative interview study is grounded in an interpretivist, phenomenologic approach focusing on individual meaning- and sense-making.^13^ The study was approved by the University of Michigan Institutional Review Board and conducted from November 1, 2021, through April 30, 2023. All participants contacted the study team, indicating interest in participation. The study team emailed the consent form for its review. After reviewing the consent form, the research staff answered any questions, received the electronic signature, and offered each participant a paper or electronically signed form. The study followed the Standards for Reporting Qualitative Research (SRQR) reporting guidelines.^14^

### Recruitment

Two groups of WWPD participated. Key informants, self-identified from a research participant registry notice (n = 16), tested 4 kit options for self-sampling. Kit options included 2 different vaginal collection devices and 2 different urine collection kits. On the basis of the feedback from the key informants, we chose the 1 vaginal and 1 urine collection kit that were most feasible and accessible for use in the pilot phase of the study. Pilot phase participants from the community (n = 40) tested 2 kits each (1 vaginal and 1 urine if they did not have a suprapubic catheter) and were recruited via collaborators already providing care to WWPD, who extended direct invitations to participate. Community-based disability advocacy organizations also advertised the opportunity to participate. Interested participants contacted our study team to be screened for eligibility. Eligible participants had a cervix, were aged 30 to 65 years, identified as having a physical disability, and could receive US mail within the contiguous 48 states. Participants could not currently have cancer, have had cervical cancer in the past, currently be pregnant, or be within 6 weeks of giving birth. If participants used a suprapubic urinary catheter, they only participated in evaluating the vaginal self-sampling devices.

### Quantitative Survey

All participants completed a quantitative survey to provide personal descriptors. They self-identified race and ethnicity, educational level, employment, current income, insurance status, marital status, health status, menopause status, health care visit frequency, opinions about accessing health care, and their cervical cancer screening priority. We assessed race and ethnicity because these characteristics are associated with different screening patterns among people without disabilities (eTable 1 in Supplement 1). We used descriptive statistics of central tendency measures, frequencies, and distributions to describe our population quantitatively using Statistica software, version 14.0 (TIBCO).^15^

### Qualitative Interviews

Participants completed a semistructured interview via telephone, lasting between 8 and 56 minutes (mean, 21 minutes). Interviews explored how individuals described their disability, their experiences with vaginal and urine self-sampling devices, barriers and facilitators to screening, and other factors that might impact their screening experience (eTable 2 in Supplement 1). The interview team underwent interviewing training in their graduate studies; all were women who did not know the study participants before the interview, and no research team members had a physical disability. The Theoretical Domains Framework informed the interview guide, particularly the domains of knowledge, skills, social identity, beliefs about capabilities, barriers or facilitators, environmental context and resources, and social influence.^16,17^ Participants received a $50 incentive to recognize their time and effort, including participating in self-sampling, completing a survey, and completing the telephone interview.

The study staff transcribed the interviews verbatim and took notes during interviews with 2 participants who declined to be recorded. Three authors (A.H.V., C.N., and E.K.H.) coded qualitative interviews to generate a thematic description of the experiences of clinician-led speculum-based screening and at-home self-sampling.^18^ We used the Dedoose platform for qualitative coding.^19^ The team developed codes inductively through coding consensus discussions and deductively using Theoretical Domains Framework domain definitions corresponding to the interview guide. One author (C.N.) coded the remaining interviews, frequently discussing them with the other 2 coders (A.H.V. and E.K.H.) to refine code definitions and ensure consistent application. Two authors (C.N. and A.H.V.) collaborated on the development and ordering of themes and the writing of findings. We present the interview excerpts in anonymized form.

## Results

A total of 56 women (mean [SD] age, 45.4 [9.1] years) participated in the study. The population was highly educated (47 [83.9%] had some college, graduated from college, or had a postgraduate degree), but only 20 (35.7%) were employed full time or part time; 45 (80.3%) declared they were living comfortably or getting by economically. All 56 (100%) had health insurance, 18 (33.3%) were never partnered, 12 (21.4%) were menopausal, 49 (87.5%) visited the physician at least once a year, and 36 (65.5%) expressed accessing health care has been primarily positive. Only 12 (21.4%) indicated cervical cancer screening was not a priority for their health, and 38 (67.8%) stated they were in good or very good health (Table). Up-to-date screening status was defined as within the last 3 years because this is the interval used in reporting the MEPS, BRFSS, and NHIS results for cervical cancer screening timeliness: 28 participants (50.0%) were up to date on their screening, 20 (35.7%) screened late, and 8 (14.3%) were not aware of the timing of their past screening event or did not screen.

**Table.** Demographic Characteristics by Cervical Cancer Screening Status for Women With Physical Disabilities

Characteristic	No. (%) of participants^a^				
	Total (N = 56)^b^	Screening (n = 53)			No screening (n = 3)
		Up to date (n = 28)	Not up to date (n = 20)	Timing unknown (n = 5)	
Age, mean (SD) [range], y	45.4 (9.1) [30-63]	42.2 (8.0) [30-59]	51.3 (7.9) [34-63]	39.0 (8.0) [35-51]	44.7 (12.9) [34-59]
Race and ethnicity	54 (100)	26 (100)	19 (100)	5 (100)	3 (100)
Asian	3 (5.6)	1 (3.8)	0	2 (40.0)	0
Asian and Black	1 (1.9)	1 (3.8)	0	0	0
Black and non-Hispanic	7 (13.0)	3 (11.5)	2 (10.5)	1 (20.0)	1 (33.3)
Canadian Indian and White	1 (1.9)	0	1 (5.3)	0	0
Hispanic and White or Hispanic alone	3 (5.6)	0	1 (5.3)	1 (20.0)	1 (33.3)
Jewish	1 (1.9)	1 (3.8)	0	0	0
Native American and Black	1 (1.9)	0	1 (5.3)	0	0
White and non-Hispanic	38 (70.4)	21 (80.8)	14 (73.7)	1 (20.0)	1 (33.3)
Multiple races	4 (7.4)	2 (7.7)	2 (10.5)	0	0
Educational level	56 (100)	28 (100)	19 (100)	5 (100)	3 (100)
Postgraduate degree	23 (41.1)	16 (57.1)	5 (26.3)	2 (40.0)	0
Graduated from college	19 (33.9)	8 (28.6)	9 (47.4)	0	1 (33.3)
Some college	5 (8.9)	1 (3.6)	1 (5.3)	2 (40.0)	1 (33.3)
Post–high school training, vocational, or technical	3 (5.4)	0	2 (10.5)	1 (20.0)	0
High school graduate or GED	6 (10.7)	3 (10.7)	2 (10.5)	0	1 (33.3)
Employment status	56 (100)	21 (100)	19 (100)	5 (100)	3 (100)
Employed full time	11 (19.6)	7 (33.3)	2 (10.5)	2 (40.0)	0
Employed part time	9 (16.1)	4 (19.0)	5 (26.3)	0	0
On disability	29 (51.8)	13 (61.9)	12 (63.2)	1 (20.0)	2 (66.7)
Homemaker or caretaker	3 (5.4)	2 (9.5)	0	0	1 (33.3)
Retired	1 (1.8)	1 (4.8)	0	0	0
Student	3 (5.4)	1 (4.8)	0	2 (40.0)	0
Current income	56 (100)	28 (100)	19 (100)	5 (100.0)	3 (100)
Living comfortably	20 (35.7)	10 (35.7)	5 (26.3)	2 (40.0)	2 (66.7)
Getting by	25 (44.6)	14 (50.0)	9 (47.4)	2 (40.0)	
Finding it difficult	7 (12.5)	3 (10.7)	3 (15.8)	0	1 (33.3)
Finding it very difficult	4 (7.1)	1 (3.6)	2 (10.5)	1 (20.0)	0
Insurance type	55 (100)	28 (100)	19 (100)	5 (100)	3 (100)
A plan you or a family member has through an employer or union	19 (34.5)	13 (46.4)	4 (21.1)	1 (20.0)	1 (33.3)
Medicaid or other state program	11 (20.0)	6 (21.4)	3 (15.8)	1 (20.0)	1 (33.3)
Medicare	22 (40.0)	7 (25.0)	12 (63.2)	3 (60.0)	0
A plan that you or a family member buys on your own	3 (5.5)	2 (7.1)	0	0	1 (33.3)
Marital status	54 (100)	26 (100)	19 (100)	5 (100)	3 (100)
Married or partnered	28 (51.9)	17 (65.4)	6 (31.6)	2 (40.0)	2 (66.7)
Single, never married or partnered	18 (33.3)	6 (23.1)	8 (42.1)	3 (60.0)	1 (33.3)
Divorced or separated	8 (14.8)	3 (11.5)	5 (26.3)	0	0
Menopause	56 (100)	28 (100)	19 (100)	5 (100)	3 (100)
Yes	12 (21.4)	3 (10.7)	8 (42.1)	0	1 (33.3)
No	40 (71.4)	24 (85.7)	8 (42.1)	5 (100)	2 (66.7)
Unsure or I don’t know	4 (7.1)	1 (3.6)	3 (15.8)	0	0
Physician visit once a year	56 (100)	28 (100)	19 (100)	5 (100)	3 (100)
Strongly agree	38 (67.9)	23 (82.1)	10 (52.6)	3 (60.0)	2 (66.7)
Agree	11 (19.6)	5 (17.9)	4 (21.1)	1 (20.0)	1 (33.3)
Neither agree nor disagree	3 (5.4)	0	2 (10.5)	0	0
Disagree	4 (7.1)	0	3 (15.8)	1 (20.0)	0
Strongly disagree	0	0	0	0	0
Experience with accessing health care has been mostly positive	55 (100)	27 (100)	19 (100)	5 (100)	3 (100)
Strongly agree	10 (18.2)	3 (11.1)	4 (21.1)	2 (40.0)	1 (33.3)
Agree	26 (47.3)	15 (55.6)	6 (31.6)	2 (40.0)	2 (66.7)
Neither agree nor disagree	12 (21.8)	4 (14.8)	7 (36.8)	1 (20.0)	0
Disagree	6 (10.9)	4 (14.8)	2 (10.5)	0	0
Strongly disagree	1 (1.8)	1 (3.7)	0	0	0
Cervical cancer screening priority	56 (100)	28 (100)	19 (100)	5 (100)	3 (100)
High priority	22 (39.3)	12 (42.9)	7 (36.8)	3 (60.0)	0
Somewhat of a priority	22 (39.3)	11 (39.3)	7 (36.8)	1 (20.0)	2 (66.7)
Not a priority	12 (21.4)	5 (17.9)	5 (26.3)	1 (20.0)	1 (33.3)
Health status	56 (100)	28 (100)	19 (100)	5 (100)	3 (100)
Very good	12 (21.4)	4 (14.3)	5 (26.3)	2 (40.0)	1 (33.3)
Good	26 (46.4)	14 (50.0)	7 (36.8)	3 (60.0)	1 (33.3)
Fair	17 (30.4)	9 (32.1)	7 (36.8)	0	1 (33.3)
Poor	1 (1.8)	1 (3.6)	0	0	0

^a^ Unless otherwise indicated.

^b^ Not all respondents answered all questions, creating missing data not noted in the Table.

Of participants who selected only 1 race category (all non-Hispanic), 3 (5.6%) were Asian, 7 (13.0%) were Black or African American, and 38 (70.4%) were White. Three (5.6%) were Hispanic or Latinx, of whom 2 indicated that they were also White. The remaining participants belonged to more than 1 racial and ethnic group; 4 (7.4%) selected multiple categories or used the write-in option to indicate racial and ethnic group membership (Asian and Black or African American, American Indian or Alaska Native and Black or African American, Canadian Indian and White, and Jewish). Two participants declined to provide both race and ethnicity information.

All participants in this study self-identified as women; hence, we report our findings as representing the experiences of WWPD. Where appropriate, we use nongendered language to recognize that although women assigned female at birth may be the majority of individuals needing cervical cancer screening, nonbinary and transgender individuals may also need this care.

### Qualitative Interviews

In the qualitative interviews, WWPD provided emotionally evocative descriptions of physical and emotional experiences of both speculum examinations and self-sampling. We collated descriptors from interview transcripts to augment the quotations presented below and demonstrate the difference in sentiment between in-office speculum examinations and at-home self-sampling (Box).

Box. **Descriptions of Cervical Cancer Screening Techniques by Women With Physical Disabilities**

**In-office speculum-based cervical cancer screening**

Awful, miserable, embarrassing, dreadful, excruciatingly/extraordinarily painful, exposure/exposed, scared/scary, vulnerability, cold, invasive, uncomfortable, humiliation, fear, murder, difficult, hard, awkward, hurt, horrific, negative, horrible, fear, major ordeal, unfavorable, stressful, not safe, tortured, rushed, anxiety, worry, negative attitudes, inconvenience, strange, injury, horribly traumatizing, daunting, apprehension, scrape, self-conscious, probing, hassle, misery

**At-home self-sampling cervical cancer screening**

Comfortable, private, simple, accessible, easier, convenient, fantastic, worlds better, control, trust, confidence, smoothly, innovative, positive, less invasive, miracle, awesome, enjoyed, wonderful, great, soft, familiar, stoked, straightforward, proud, flexible, excited, inviting, routine, intuitive, excellent, reasonable, discrete, feasible, useful, beneficial, accessible, manageable, important, safe

#### Physical and Emotional Experiences of In-Office Screening

The qualitative interviews revealed that 49 of 53 women (92.5%) described negative prior experiences with clinician-led speculum examinations (3 had no previous experience), highlighting disability-specific factors that impacted their experiences. One significant contributor was the built environment of the clinic setting and the default position for speculum exams: “Like for me, trying to get my feet in the stirrups, I can’t do that…because my hips are going to dislocate to the side and…my ankle in the stirrup is probably going to go out too.” In addition to the built environment, 44 women (88.7%) described the in-office screening as inconvenient in ways that were exacerbated by disability-specific considerations, such as needing to carefully choose clothing that was easy to get in and out of and bringing items such as a dressing stick and sock aid to get dressed after the examination.

When our participants went to the clinic for clinician-led screening, several reported feeling that they were treated differently by health care staff due to their disability or needed accommodations. One participant described, “I prefer to go on my own because it forces the doctors to talk to me. You have someone else in the room, when you have a physical disability, they’re just going to talk to the other person.” Another interviewee described an experience where she wondered if the physician performing the examination was hurting her on purpose: “He couldn’t understand that I could not like lift my legs very high at all. …He was thoroughly disgusted…And it was very painful, very painful. And I don’t know if he did that because he was mad…And I know after I got the results from his nurse, I was supposed to go back in and get a colposcopy and I never did…it’s been out of fear. Not really out of fear of the result, but fear of having to go through that humiliation again.” As this interviewee described, this experience was so upsetting that she did not return for additional testing even after receiving an abnormal screening result. Importantly, she clarified that her fear of seeking follow-up care was not because of fear of a cancer diagnosis but because of the humiliation she experienced from her clinician.

#### Physical and Emotional Experiences of At-Home Self-Sampling

In contrast, all 56 participants described in the interviews how self-sampling was both comfortable and convenient. The ability to screen at home eliminated some common barriers to in-office screening, such as difficulty with transportation and time constraints and added planning related to their needed accommodations. As one woman stated, “I liked [self-sampling] better…Because you know, going to the doctor is a major ordeal for me. You know, getting ready, my oxygen together, packing the diapers, and getting there, and walking whatever distance I’ve got to walk.”

In addition to being able to collect the sample themselves at home, where they have a comfortable, accessible set-up and assistance if needed, many women felt that using the self-sampling kits was similar to tasks they had performed before (eg, using tampons), which made the experience feel easier and more familiar. Interviewees also described similarities to pregnancy tests, COVID-19 tests, and home-based colon cancer screening. As one woman stated, “I do colon cancer screening at home, so why wouldn’t I do this at home?” Overall, 8 participants (14.3%) sought assistance with the vaginal self-sampling device from a partner, family member, or caregiver, 2 of whom (3.6%) also sought assistance with the urine collection kit.

For women who already face disabling environments in their day-to-day lives, providing the option to choose when and where to screen for cervical cancer allows them control over their health: “I wouldn’t have to go to the doctor to [get screened]. I could just do it myself. So, [I] like having the control over that. Especially with being disabled you don’t have control over a lot.”

#### Screening Preferences and Impact on Screening Rates

When asked during the qualitative interviews, 37 of 56 WWPD (66.1%) believed that self-sampling would change the likelihood that they would obtain on-time screening in the future. Although the remaining 19 WWPD stated they would still opt for the in-office examination, they reported that they would recommend self-sampling to others.

When asked to describe how self-sampling would impact the likelihood of them getting screened in the future, 1 woman responded by saying: “I’ve been really hesitant about going in and getting one…But I would do it more on a regular basis if I was able to do it at home.” In further explaining how a self-sampling option would increase their likelihood of getting screened, women gave both disability-specific and non–disability-specific reasons: “I didn’t have to drive an hour and a half to do it. I didn’t have to wait in the lobby to be called. I didn’t have to get up on the table, try to undress. I didn’t have strange people looking at my private area…I didn’t have to have my leg smacking down, causing an injury. And then the driving home. That’s why I don’t do it. This [self-sampling], I would do.”

#### Considerations for Expanding Self-Sampling

Participants described that self-sampling could relieve disability-specific barriers to cervical cancer screening, and they expressed excitement that research was happening to improve experiences for WWPD. Participants also explained that the benefits of self-sampling could extend to women more broadly: “I think most women are like me where it’s not something they look forward to…And if it’s this easy to do it at home versus feeling so exposed and inconvenienced at times by going to a doctor’s office, I don’t know who wouldn’t choose that option.” Another participant highlighted that self-sampling could also benefit nonbinary people and transgender men who need screening: “There are a lot of people who have vaginas that don’t identify as women. And I’m sure they would be more likely to get screened if there was something available they could do in their home.” Finally, although this research focused on WWPD, 1 interviewee explained that the availability of self-sampling methods could ease screening barriers for individuals with intellectual and developmental disabilities as well: “My sister is special needs, and this urine kit is something that we could do with her because we cannot take her in for a Pap smear, she cannot get through that experience.”

Participant responses also identified opportunities to address misconceptions around the accuracy of self-sampling compared with speculum examinations and to provide education about the correct way to obtain a self-sample. For example, 1 interviewee wondered if different accuracy rates were a reason why she had never been offered a urine testing option before: “So now, again, the accuracy, I don’t know yet…Because first of all, I’ve never had a urine test to test for it, and if that’s the case [that urine screening is accurate], why have I been getting screwed open on a metal speculum for years when I could have just peed in a cup?” Other interviewees cited a lack of confidence in their ability to collect an adequate sample, such as 1 woman who stated, “Well, I think that going to a doctor and doing a Pap smear, for me, personally, is the best option. Because I’m not a doctor and I’m not a professional, and doctors know what they’re doing. Like, I was fearful…I’m trying to follow the instructions, but did I do them right?” This participant did not cite disability-specific reasons for concern that she may not collect the sample correctly; rather, she expressed a general concern about user error as someone who is not a medical professional.

## Discussion

Individuals with physical disabilities face unique barriers to cervical cancer screening, which may lead to them being disproportionately underscreened.^2^ Most women in our study found the use of at-home self-sampling kits more favorable to in-office speculum-based screening and believed that having the option to use one of these kits would increase the likelihood of them getting screened in the future. Moreover, providing the option to try several different types of kits allowed participants to choose options compatible with their level of dexterity, use of a urinary catheter, and other disability-specific considerations. However, although access to self-sampling kits may help WWPD participate in screening in ways that are accessible and safe, self-sampling will not alleviate the lack of clinician training around disability health. As our interviewees described poignantly, clinician ableism—evidenced through negative interactions, care that is not disability competent, and inaccessible clinic spaces—is a significant factor shaping WWPDs’ experience with and participation in cervical cancer screening.

Listening to participants describe experiences with different screening modalities helps us understand the barriers to equitable screening and see the potential of mitigating these barriers through at-home self-sampling. Within the medical model, framings of disability locate the problem within a disabled person’s body, emphasizing their physical, mental, or intellectual limitations. Adopting the perspective of the social model of disability^20^ encourages us to examine features of the built and social environment and to ask whether those features contribute to disabling someone by limiting their participation in everyday activities. The participants’ accounts of inaccessible clinical environments and clinician ableism and their desire for greater autonomy over their health care demonstrate good support for self-sampling kits to be used for cervical cancer screening for WWPD. Self-sampling methods can contribute to creating an enabling world for people with disabilities and for those of us who are temporarily able-bodied.^21^

Eliciting interviewees’ perspectives helps us understand opportunities for education and support so that all people using self-screening devices can use them confidently. Although education about correctly self-collecting a sample and the accuracy of the screening result are important across the board, this information may be essential for individuals facing screening disparities. Clinicians must provide accurate, evidence-based information to their patients regarding the role of screening examinations in gynecologic care, as well as information about alternative forms of cervical cancer screening, to reduce barriers to on-time cervical cancer screening.

### Limitations

This study has some limitations. First, our study recruitment took place through 2 pathways: a community organization and individual clinicians. One of the physicians who referred participants to our study is a specialist in providing gynecologic care for WWPD. Although this allowed us an opportunity to learn from some WWPD about what accessible care can look like, this could be viewed as a limitation of our design in that some participant interviews did not allow us to understand the typical experience for WWPD. A second limitation of our study design is that, although we recruited a racially diverse sample, our interview guide focused primarily on participants’ experiences as cisgender women with disabilities. This focus allowed us to understand some intersections between disability and gender but not the intersection of these 2 identities with race and ethnicity. The opportunity to understand how racial and ethnic group membership intersects with gender and disability status to shape cervical cancer screening experiences motivates our future work in this area. Third, the level of hand and arm dexterity required to use self-sampling kits may vary by kit type. Although the participants in our study could try out a range of kits and we did note the role of dexterity in self-sampling experience, hand and arm agility was not a formal inclusion or exclusion criterion for participation eligibility.

## Conclusions

In this qualitative study of WWPD, we found that this group preferred self-sampling as an alternative to in-office speculum-based cervical cancer screening, which may increase screening participation. Although our study focused on the experiences of WWPD, our findings align with the positive experiences of self-sampling among other groups of women,^16^ suggesting the broad appeal of self-sampling devices. Whole-population cervical cancer screening rates for WWPD remain below target.^22^ Because the US Food and Drug Administration has approved self-sampling for cervical cancer screening, lessons learned from our study may inform education and promotion efforts to all people as the medical field makes this change in collection technique.

## Notes

Supplement 1. **eTable 1.** Quantitative Survey **eTable 2.** Interview Script

Supplement 2.
Data Sharing Statement

## References

[1] NORC at the University of Chicago. Only 14% of cancers are detected through a preventive screening test. Accessed March 12, 2024. https://www.norc.org/content/dam/norc-org/pdfs/State-Specific%20PCDSs%20chart%201213.pdf

[2] Drew JA, Short SE. Disability and Pap smear receipt among U.S. Women, 2000 and 2005. Perspect Sex Reprod Health. 2010;42(4):258-266. doi:10.1363/4225810 21126302 PMC4181604

[3] Lagu T, Hannon NS, Rothberg MB, . Access to subspecialty care for patients with mobility impairment: a survey. Ann Intern Med. 2013;158(6):441-446. doi:10.7326/0003-4819-158-6-201303190-00003 23552258

[4] Kalpakjian CZ, Kreschmer JM, Slavin MD, . Reproductive health in women with physical disability: a conceptual framework for the development of new patient-reported outcome measures. J Womens Health (Larchmt). 2020;29(11):1427-1436. doi:10.1089/jwh.2019.8174 32429740 PMC7703246

[5] Milligan MS, Neufeldt AH. The myth of asexuality: a survey of social and empirical evidence. Sex Disabil. 2001;19:91-109. doi:10.1023/A:1010621705591

[6] Nosek MA, Howland C, Rintala DH, Young ME, Chanpong GF. National study of women with physical disabilities. Sex Disabil. 2001;19:5-40. doi:10.1023/A:1010716820677

[7] Houtenville A, Bach S, Paul S. Annual Report on People with Disabilities in America: 2023. Institute on Disability. University of New Hampshire; 2023.

[8] Meites E, Gee J, Unger E, Markowitz L. Human Papillomavirus. 14th ed. Epidemiology and Prevention of Vaccine-Preventable Diseases; 2021:chap 11. Accessed January 7, 2025. https://www.cdc.gov/pinkbook/hcp/table-of-contents/index.html

[9] Bacopoulou F, Karakitsos P, Kottaridi C, . Genital HPV in children and adolescents: does sexual activity make a difference? J Pediatr Adolesc Gynecol. 2016;29(3):228-233. doi:10.1016/j.jpag.2015.08.010 26342734

[10] Polman NJ, Snijders PJF, Kenter GG, Berkhof J, Meijer CJLM. HPV-based cervical screening: rationale, expectations and future perspectives of the new Dutch screening programme. Prev Med. 2019;119:108-117. doi:10.1016/j.ypmed.2018.12.021 30594536

[11] Iezzoni LI, Rao SR, Ressalam J, . Physicians’ perceptions of people with disability and their health care. Health Aff (Millwood). 2021;40(2):297-306. doi:10.1377/hlthaff.2020.01452 33523739 PMC8722582

[12] Iezzoni LI. Cancer detection, diagnosis, and treatment for adults with disabilities. Lancet Oncol. 2022;23(4):e164-e173. doi:10.1016/S1470-2045(22)00018-3 35358465

[13] Hanson JL, Balmer DF, Giardino AP. Qualitative research methods for medical educators. Acad Pediatr. 2011;11(5):375-386. doi:10.1016/j.acap.2011.05.001 21783450

[14] O’Brien BC, Harris IB, Beckman TJ, Reed DA, Cook DA. Standards for reporting qualitative research: a synthesis of recommendations. Acad Med. 2014;89(9):1245-1251. doi:10.1097/ACM.0000000000000388 24979285

[15] TIBCO Software Inc. Data Science Workbench, version 14. Accessed March 12, 2024. http://tibco.com

[16] Atkins L, Francis J, Islam R, . A guide to using the Theoretical Domains Framework of behaviour change to investigate implementation problems. Implement Sci. 2017;12(1):77. doi:10.1186/s13012-017-0605-9 28637486 PMC5480145

[17] Haro E, Butcher EA, Alves ML, El Khoury C, Vinson A, Harper DM. Barriers to cervical cancer screening and satisfaction with self-sampling among Black women in Michigan: a mixed methods study. Med Res Arch. 2024;12(4). doi:10.18103/mra.v12i4.5209 38818307 PMC11138408

[18] Kiger ME, Varpio L. Thematic analysis of qualitative data: AMEE Guide No. 131. Med Teach. 2020;42(8):846-854. doi:10.1080/0142159X.2020.1755030 32356468

[19] Dedoose, version 9.2.12 [computer software]. SocioCultural Research Consultants; 2024. Accessed July 19, 2024. http://www.dedoose.com

[20] Marks D. Models of disability. Disabil Rehabil. 1997;19(3):85-91. doi:10.3109/09638289709166831 9134350

[21] Davis LJ. Enforcing Normalcy: Disability, Deafness, and the Body. Verso; 1995.

[22] Office of Disease Prevention and Health Promotion, US Department of Health and Human Services. Increase the proportion of females who get screened for cervical cancer. In: *Healthy People 2030*. US Dept of Health and Human Services. Accessed July 19, 2024. https://health.gov/healthypeople/objectives-and-data/browse-objectives/cancer/increase-proportion-females-who-get-screened-cervical-cancer-c-09

