# Supplementary material for: Cervical Cancer Screening in Women With Physical Disabilities
Source: JAMA Netw Open. 2025 Jan 29;8(1):e2457290. doi: 10.1001/jamanetworkopen.2024.57290 (PMC11780472; doi:10.1001/jamanetworkopen.2024.57290)
Supplement: Supplement 1. — eTable 1. Quantitative Survey eTable 2. Interview Script [file jamanetwopen-e2457290-s001.pdf]

Supplementary Online Content

Vinson AH, Norrid C, Haro EK, et al. Cervical cancer screening in women with physical disabilities. *JAMA Netw Open*. 2025;8(1):e2457290. doi:10.1001/jamanetworkopen.2024.57290

eTable 1. Quantitative Survey

eTable 2. Interview Script

This supplementary material has been provided by the authors to give readers additional information about their work.

© 2025 Vinson AH et al. *JAMA Network Open*.

eTable 1. Quantitative Survey

Record I	Text
MISSH3	Key informant (16)<br>Full study group (40)
Age	30-45<br>46-65<br>Other
How old are you?	Number
Do you have a cervix?	Yes/no
Have you had a hysterectomy to remove your uterus?	Yes/no
Are you currently pregnant?	Yes/no
Are you currently diagnosed with cancer – not including cancer in remission or non-melanoma skin cancer?	Yes/no
Do you live in the contiguous US?	Yes/no
Do you identify as having a physical disability that involves any kind of mobility impairment (for example, your ability to walk, control your muscles, or use your hands)?	Yes/no
Are you limited in any way because of this mobility impairment?	Yes/no
Have you been limited for more than 6 months	Yes/no
How old were you when your moblity impairment began?	number
Because of any impairment or health problem do you need the help of other persons with your PERSONAL CARE needs, such as eating, bathing, dressing, or getting around the house?	Yes/no
Because of any impairment or health problem, do you need the help of other persons to handle your ROUTINE needs, such as everyday household chores, doing necessary business, shopping, or getting around for other purposes?	Yes/no
Do you regularly use a urinary catheter?	Yes/no
What is your age?	Number

© 2025 Vinson AH et al. *JAMA Network Open*.

Which best describes your race? [Mark all that apply].	• Asian<br>• American Indian or Alaska Native<br>• Black or African American<br>• Middle Eastern or North African<br>• Native Hawaiian or Other Pacific Islander<br>• White or Caucasian<br>• Some other race or origin, please specify
Some other race or origin	Text
Are you of Hispanic, Latino, or Spanish ethnicity?	Yes/No
Do you consider yourself to be	• A woman<br>• A man<br>• other
What is the highest level of schooling you completed?	• Less than 8 years<br>• 8-11 years<br>• High school graduate (or obtained GED)<br>• Post-high school training other than college (vocational or technical training)<br>• Some college<br>• Graduated from college<br>• Postgraduate degree
Which of the following best characterizes your current occupational status?	• employed full time<br>• employed part-time<br>• unemployed<br>• housemaker or caretaker<br>• student<br>• retired<br>• on disability<br>• otherwise unable to work (please explain)<br>• other (please specify)
Which of the following best describes your feelings about your current household income?	• living comfortably on current income<br>• getting by on current income<br>• finding it difficult on current income<br>• finding it very difficult on current income
Which of the following best describes your current relationship status?	• married or partnered<br>• single, never married or partnered

© 2025 Vinson AH et al. *JAMA Network Open*.

	• widowed<br>• divorced or separated
In general, would you say your health is	• excellent<br>• very good<br>• good<br>• fair<br>• poor
Do you have any kind of health insurance or health care coverage?	Yes/no
Have you entered menopause? (at least one year without a period)?	Yes/no/unsure
I visit my doctor at least once a year for a physical, check-up, or recommended health screening	• strongly agree<br>• agree<br>• neither agree nor disagree<br>• disagree<br>• strongly disagree
My experiences accessing healthcare (e.g., going to the doctor) have been mostly positive	• strongly agree<br>• agree<br>• neither agree nor disagree<br>• disagree<br>• strongly disagree
When was the last time you were screened for cervical cancer?	• less than 3 years ago<br>• between 3-5 years ago<br>• between 6-10 years ago<br>• more than 10 years ago
How would you prioritize cervical cancer screening in terms of your overall healthcare needs right now?	• it’s a high priority for me<br>• it’s somewhat of a priority for me<br>• it’s not a priority for me

© 2025 Vinson AH et al. *JAMA Network Open*.

eTable 2. Interview Script

Introduction

In this interview, we would like to find out what you thought about using the home testing kits – how it was for you and what feedback you might have about how to make the experience better.

First, I'll begin with some background questions. Then we'll go through the kits you used individually and get feedback about each once. To finish we'll end with a few more questions about whether or not these kits change the way you feel about cervical cancer screening.

So, a lot of questions will be focused on each of the kits you tried but there will also be a lot of questions about you in general, to add some context to your answers. If there are any questions that you don't want to answer just say "skip" and we'll move on to the next one. And if there are any questions that you don't understand please let me know and I can ask it in a different way.

Any questions before we begin?

1. In this study, we are talking to women who all identify as having some type of physical disability or mobility impairment. However, this can look very different for each person so, to start, we would like to get a sense of how you define this for yourself. In your own words, how would you describe your physical disability or mobility impairment?

2. Have you been given a specific diagnosis? [if not already mentioned]

3. How long have you had your disability / mobility impairment? [if not already mentioned]

4. If you had to choose, how would you rate your disability / mobility impairment: Would you say it is mild, moderate, or severe?

    4a. What made you choose that level?

5. Before this study, had anyone ever talked to you about cervical cancer screening?

    5a. Who?

6. As far as you know, have you ever been screened for cervical cancer before?

    6a. [If YES] Can you tell me a little bit about what that experience was like for you?

7. Okay so next we'll go into feedback about the kits. Before we talk about each kit individually, we first want to know how you felt about this experience overall. How comfortable were you being asked to provide vaginal and urine samples at home?

8. How similar was using the kits to other things that you normally do? For example, what do you usually do for your periods?

9. Okay, and now we'll go through the kits one by one to get your feedback. So you were sent [X] different kits and asked to use them in a particular order, is that correct?

    9a. [If NO] Can you tell me about what instructions were included in the package you received?

9b. [If YES] Were you able to collect a sample using all [X] kits?

9c. [If NO] Can you tell me a bit what happened and whether or not you were able to use at least one of the kits?

10. [If YES] Okay great, let's start by talking about the kit you used first, that was the ________ kit correct?

11. Were you able to use the kit on your own or did you have someone help you collect the sample?

12. How easy or challenging was it to actually use this kit?

13. Was there anything about this kit that you particularly liked or didn't like?

14. How were the instructions?

15. Is there anything we could have changed or done differently that would have made things easier with this kit?

[Repeat the above group of questions for each kit the participant tested]

16. Okay now, thinking about and comparing all of the kits you used: If you could choose, which kit would you prefer to use if you were to get screened again?

17. Why was that one your favorite? [if not already mentioned]

18. How do you think these kits compare to going to the doctor and having a Pap smear?

19. Any questions for me before we wrap up?

© 2025 Vinson AH et al. *JAMA Network Open*.
