# Supplementary material for: Cervical Cancer Screening in Women With Physical Disabilities
Source: JAMA Netw Open. 2025 Jan 29;8(1):e2457290. doi: 10.1001/jamanetworkopen.2024.57290 (PMC11780472; doi:10.1001/jamanetworkopen.2024.57290)
Supplement: Supplement 2. — Data Sharing Statement [file jamanetwopen-e2457290-s002.pdf]

Data Sharing Statement

Vinson. Cervical Cancer Screening in Women With Physical Disabilities. *JAMA Netw Open*. Published January 29, 2025. doi:10.1001/jamanetworkopen.2024.57290

Data
Data available: No

Additional Information
Explanation for why data not available: interviews of vulnerable people with disabilities discussing sensitive issues around the speculum exam
